# Insomnia Severity in Adults with Autism Spectrum Disorder is Associated with sensory Hyper-Reactivity and Social Skill Impairment

**DOI:** 10.1007/s10803-019-03891-8

**Published:** 2019-02-09

**Authors:** Vanessa D. Hohn, Danielle M. J. de Veld, Kawita J. S. Mataw, Eus J. W. van Someren, Sander Begeer

**Affiliations:** 1 Section Clinical Developmental Psychology, Faculty of Behavioural and Movement Sciences, Vrije Universiteit, Van der Boechorststraat 1, 1081 BT Amsterdam, The Netherlands; 2 Department of Sleep and Cognition, Netherlands Institute for Neuroscience, An Institute of the Royal Netherlands Academy of Arts and Sciences, Amsterdam, The Netherlands; 3 Departments of Psychiatry and Integrative Neurophysiology, Center for Neurogenomics and Cognitive Research (CNCR), Amsterdam Neuroscience, Vrije Universtiteit Amsterdam, Amsterdam UMC, Amsterdam, The Netherlands; 4 Departments of Integrative Neurophysiology and Psychiatry, Neuroscience Campus Amsterdam, VU University and Medical Center, Amsterdam, The Netherlands

**Keywords:** Autism spectrum disorder, Adults, Insomnia, Sleep problems, Sensory hyper-reactivity, Social skills

## Abstract

Insomnia is a common source of distress in adults with autism spectrum disorder (ASD). Two characteristics of ASD could be relevant to insomnia complaints by hampering the entrainment of a circadian sleep-wake rhythm. First, sensory hyper-reactivity could lead to bright light avoidance and thus affect photoperiodic input to the circadian system. Second, impaired social skills complicate the establishment of a social interactions and thus affect scheduled social-behavioral input to the circadian system. We investigated the association of insomnia severity with sensory reactivity and social skills in 631 adults (18–65 years) with ASD. Results revealed positive associations of insomnia severity with general and visual sensory hyper-reactivity and with impairment of social skills. The findings warrant further studies which (1) directly assess whether a suboptimal functioning of the biological clock underlies these associations and (2) identify other factors that could contribute to observed sleep problems.

Autism spectrum disorder (ASD) is a lifelong neurodevelopmental disorder affecting around 0.6–1.7% of the population worldwide (Baio et al. 2018; Kogan et al. 2009; Matson and Kozlowski 2011). Although individuals with ASD differ widely in terms of symptom severity, intelligence and functional ability, overarching symptoms include deficits in social communication and interaction as well as restricted and repetitive behaviors and interests, and sensory responsiveness (American Psychiatric Association 2013). Often, these primary symptoms are associated with secondary comorbid conditions. Of these, sleep problems figure prominently with prevalence rates ranging from 50 to 80% in children with ASD (Mannion et al. 2014). Typically, symptoms include delayed sleep onset, impaired sleep maintenance, early awakenings, and subsequent daytime fatigue (Krakowiak et al. 2008; Mayes and Calhoun 2009; Richdale and Schreck 2009), which closely overlap with the diagnosis of insomnia specified by the International Classification of Sleep Disorders (ICSD; American Academy of Sleep Medicine 2014). These sleep problems have been shown to persist from child- to adulthood (Baker and Richdale 2015; Goldman et al. 2011; Limoges et al. 2005; Tani et al. 2003) leading to adverse consequences for health and daily functioning throughout life (Goldman et al. 2009; Limoges et al. 2013; Matson et al. 2008; May et al. 2015; Richdale et al. 2014). Moreover, the relationship between primary symptoms and sleep problems may be bidirectional (worsening of primary symptoms leads to increasing sleep problems and vice versa) creating a vicious circle (Adams et al. 2014). Undoubtedly, this makes interventions indispensable. Yet, the development of a targeted treatment critically depends on knowledge regarding the etiology of ASD related sleep problems in children and adults, which, to date, remains limited.

It has been proposed that a disturbance of circadian rhythms could contribute to poor sleep in ASD (Baker and Richdale 2017; Bourgeron 2007; Patzold et al. 1998; Richdale and Schreck 2009). The term circadian rhythm refers to biological processes displaying an endogenous oscillation of about 24 h as can be seen for several bodily functions, including the sleep-wake cycle, temperature control, thirst, eating patterns, hormone production, and cell regeneration (Panda et al. 2002). Following this line of reasoning, sleep onset difficulties could be linked to phase delay, sleep maintenance problems to an instability of the circadian clock establishing the rhythm and early awakenings to a phase advance (Guénolé et al. 2011). The establishment of these rhythms is orchestrated by the *suprachiasmatic nucleus* (SCN), which functions as an internal biological clock. The activity of the SCN synchronizes to several types of environmental and behavioral 24-h rhythms which is referred to as entrainment. The environmental light-dark cycle has the strongest entraining effect. Even without a functional SCN, light-dark cycles can induce a rhythm in core body temperature (Scheer et al. 2005) that in turn supports the sleep-wake rhythm (Van Someren 2004). In addition to this primary photoperiodic mechanism, social cues derived from the interaction with other individuals and a fixed social schedule in home, school and work settings have been shown to contribute to the entrainment of circadian rhythms (Grandin et al. 2006; Mistlberger and Skene 2004).

Interestingly, both photoperiodic and social input sources of the circadian system are closely related to and hence may be affected by two core characteristics of the ASD symptomatology, namely alternations in sensory reactivity and deficits in social interaction. Although the nature of ASD-related sensory atypicalities varies substantially, hyper-reactivity across modalities is a common finding, which is often associated with distress in response to stimulation within the respective modality (Baron-Cohen et al. 2009). Consequently, situations which entail pronounced stimulation, e.g. in forms of bright light or noise, are avoided or attenuated by means of sunglasses or headphones (Davidson and Henderson 2016), thus reducing photoperiodic input. Likewise, the impairment of social skills observed in ASD often hampers the establishment of a social schedule which could aid entrainment of the sleep wake cycle. While children with ASD might strongly benefit from familial, environmental (e.g. school), and social factors (e.g. hobbies, play dates arranged by parents) helping to maintain an appropriate sleep-wake schedule, adults tend to be more flexible in the scheduling of their daily routines such as sleep timing. Social interactions and a structured work setting are two main factors which can restrict this flexibility within healthy boundaries. However, both the amount of social interactions and employment rates have been shown to be reduced in adults with ASD when comparing them to the general population or other disabilities (Baker and Richdale 2017; Baker et al. 2018; Hedley et al. 2017; Orsmond et al. 2004). Therefore, it seems reasonable to argue that, by reducing entrainment of the biological clock, sensory hyper-reactivity and impaired social skills may both contribute to insomnia in ASD.

Supporting this line of reasoning, associations between sensory responsivity and/or social skills with sleep quality have been reported. Increased sensory responsiveness to internal as well as external responses, for example, is a key feature of insomnia (Te Lindert et al. 2018; Wei et al. 2016). Further, individual differences in light sensitivity have been shown to affect sleep in adolescents (Van der Meijden et al. 2016). With respect to ASD, links between increased sensory responsiveness, decreased social skills and the presence of sleep problems have been reported previously in children and adolescents (Hollway et al. 2013; Liu et al. 2006; Mazurek and Petroski 2015; Reynolds et al. 2011; Tzischinsky et al. 2018). Interestingly, the effect of sensory responsiveness varied depending on the modality investigated with strongest associations in the tactile and auditory domain (Hollway et al. 2013; Tzischinsky et al. 2018) as well as the age group investigated with stronger effects for older children (Mazurek and Petroski 2015). Studies examining this relationship in adults are still lacking, however. Yet, they are highly relevant given (1) the prevalence of and psychological strain caused by sleep problem in this population as well as (2) the fact that the establishment of a mature sleep-wake cycle is a developmental phenomenon indicating that mechanisms underlying sleep problems in distinct age groups may differ substantially (Jenni and Carskadon 2007).

The current study aimed at critically examining the link between sensory responsiveness, social skills, and insomnia in adults with ASD to advance the understanding of potential underlying mechanisms. Based on the findings in pediatric populations, low social skills and increased sensory responsivity were hypothesized to predict the severity of insomnia symptoms displayed by adults with ASD. Further, we expected the strength of the relationship between sensory responsivity and insomnia severity to vary depending on the sensory modality addressed.

## Method

### Procedure

Data were obtained from the Netherlands Autism Register (NAR), which is a longitudinal register including approximately 2000 individuals with ASD. The NAR has been established by the Dutch Association for Autism (Nederlandse Vereniging voor Autisme; NVA) in collaboration with the Vrije Universiteit Amsterdam (VU) and operates primarily through online questionnaires covering various domains of functioning as well as demographical data. The Medical Ethical Committee of the Vrije Universiteit Amsterdam approved this research. For the present study, individual responses given to three measures of interest during an online survey in 2015 were analyzed.

### Sample

The age range was restricted to 18 through 65 years during the respective time point resulting in the following sample characteristics: Data were obtained from 631 individuals with autism (M_age_ = [42.62(12.21)], 48% males). Men from this sample were older than women (45.49 vs. 39.98 years respectively, *t*(629) = 5.81, *p* < .001). With respect to the presence of sleep problems, the mean score obtained on the Insomnia Severity Index (ISI, Morin 1993) was 9.50 (*SD* = 6.01), which is indicative of subthreshold insomnia. For an overview of the sample composition with respect to other variables of interest see Tables 1 and 2.

**Table 1:** Descriptive statistics of covariates and their relationship with insomnia severity

	*M*	*SD*	Min	Max	*N*	*t*	*r*
Covariates							
Sex							
Female					328	− 3.51 (*p* < .001)	
Male					303		
Medication intake							
Yes					253	1.76 (*p* = .08)	
No					378		
IQ							
IQ < 116					240		− .10 (*p* = .02)
IQ 116–130					258		
IQ > 130					133		
Age	42.62	12.21	18.05	64.93	631		− 01 (*p* = .10)
18–25	21.46	2.02	18.05	24.83	60		.20 (*p* = .13)
25–45	35.16	5.60	25.17	44.93	271		.12 (*p* = .19)
45–65	53.51	5.29	45.01	64.93	300		− .15 (*p* = .01)

The influence of sex and medication intake on ISI (insomnia severity index) scores was examined using dependent sample t-tests. The impact of age and intelligence was evaluated by means of Pearson correlations. Variables significant at a level of alpha ≤ .1 were retained and entered as covariates during further analyses

**Table 2:** Descriptive statistics of predictors and insomnia severity

	*M*	*SD*	Min	Max	*N*
Predictors					
SPQ total score	43.40	15.59	3.00	93.00	550
SPQ smell	13.79	5.73	0.00	30.00	550
SPQ vision	7.50	3.33	0.00	18.00	550
SPQ hearing	7.21	3.04	0.00	15.00	550
SPQ touch	10.66	4.77	0.00	26.00	550
SPQ taste	4.24	2.22	0.00	11.00	550
Social skills	21.63	3.93	7.00	28.00	549
Outcome measure					
Insomnia Severity Index	9.50	6.01	0.00	28.00	623

*SPQ* Sensory Perception Quotient

### Measures

#### Insomnia Severity Index

Insomnia symptoms were measured using the Insomnia Severity Index (ISI; Morin 1993), which consists of seven self-report items assessing the nature, severity and impact of insomnia within an individual. Responses to single items are given on a five-point Likert scale adjusted to the respective item, with “0” indicating that a symptom is not present and “4” that a symptom is very pronounced or worrisome. Subsequently, single scores are summed to a total score ranging from 0 to 28. While total scores between 0 and 7 indicate the absence of insomnia, scores from 8 to 14 suggest sub-threshold insomnia, followed by moderate insomnia (15–21), and severe insomnia (22–28). Adequate internal consistency and high concurrent validity and high internal (Cronbach’s alpha = .87) have been established (Bastien et al. 2001; Morin et al. 2011).

#### Sensory Perception Quotient: Short Version

The short version of the Sensory Perception Quotient (SPQ; Tavassoli et al. 2014) consists of 35 items and measures basic sensitivity in the five main sensory modalities including smell, vision, hearing, touch, and taste. Items are worded to identify hypersensitivity as well as hyposensitivities, hence reducing potential response biases. Responses are given on a four-point Likert scale (“0 = strongly agree” to “3 = strongly disagree”). The total score is obtained by summing the responses to all 35 items leading to a total score range of 0–105 with a lower score indicating higher sensory sensitivity. Subscale scores for each modality are obtained by summing the responses to corresponding items. As the number of items per scale varies (smell: 10, score range 0–30; vision: 6, score range 0–18; hearing: 5, score range 0–15; touch: 10, score range 0–30; taste: 4, score range 0–12) subscale scores cannot be compared between modalities. However, within a modality lower scores again indicate higher sensory reactivity. Good reliability and validity have been reported (Tavassoli et al. 2014). Likewise, high internal consistency was obtained in the present study for both the SPQ total score (Cronbach’s alpha = .93), as well as modality-specific subscales (smell: Cronbach’s alpha = .84, vision: Cronbach’s alpha = .71, hearing: Cronbach’s alpha = .73, touch: Cronbach’s alpha = .80, taste: Cronbach’s alpha = .84).

#### Autism Spectrum Quotient-28

Subscale social skills. Social skill impairments were assessed through the social skills subscale of the Autism Spectrum Quotient-28 (AQ-28; Hoekstra et al. 2011). The AQ-28 is a short version of the AQ questionnaire (Baron-Cohen et al. 2001), which is a self-report tool including 25 ASD-congruent and 25 ASD-incongruent questions. By means of exploratory and subsequently confirmatory factor analysis, the 50 original items were reduced to 28 items (Hoekstra et al. 2011). These can be assigned to five distinct factors, assessing difficulties with social skills, attention switching and imagination, as well as a preference for routine, and a fascination for numbers/patterns. For each question responses are given on a four-point Likert scale ranging from “1 = definitely agree” to “4 = definitely disagree”. Final scores of the subscale social skills consisting of eight questions range from 1 to 32, with higher scores indicating full endorsement of autistic characteristic, namely pronounced impairments with respect to social skills. Adequate psychometric properties have been reported (Bishop et al. 2004; Hoekstra et al. 2011; Sonié et al. 2013). High internal consistency was obtained for the social skills subscale in the present study as well (Cronbach’s alpha = .84).

### Statistical Analyses

#### Primary Analyses

Based on reported effects of medication, biological sex, intelligence, and age on sleep behaviors in children and adolescents with ASD (Liu et al. 2006; Mannion et al. 2014) the presence of similar relationships was examined to identify potential confounding variables. To determine whether individuals taking medication during the assessment differed from those without medication with respect to their ISI total score and to examine sex related effects, two independent sample t-tests were conducted. The impact of age and intelligence on ISI scores was evaluated by means of Pearson correlations. Variables significant at a level of alpha ≤ .1 were retained and entered as covariates during further analyses.

#### Hierarchical Multiple Linear Regression Analyses

Two hierarchical multiple linear regression analyses (HMLR) were applied to examine the relationship between the two predictors, i.e. sensory responsivity and social skills, and sleep problems while controlling for confounding effects of covariates. Significant covariates identified during the primary analyses were entered in the first block, followed by the predictors. While a first HMLR examined the influence of the total SPQ score, the five subscale values representing the main sensory modalities were entered into a second HMLR model. Alpha was set to 0.05.

## Results

Descriptive statistics of all variables included in the analyses can be found in Tables 1 and 2. The mean ISI score in the present sample was 9.50 (*SD* = 6.01), which is indicative of subthreshold insomnia and higher than means reported for the general population ranging between 1 and 7 (Gerber et al. 2016; Tang et al. 2007), but lower than values obtained from insomnia patients ranging between 17 and 20 (Bastien et al. 2001; Castronovo et al. 2016). When looking at the different ISI categories, 46% of the sample fell within the first category (absence of insomnia) followed by 34% with subthreshold insomnia and 17% with moderate insomnia. Only 3% displayed signs of severe insomnia.

Primary analyses revealed that each of the covariates had a significant impact on the ISI total score at *p* ≤ .1 (sex: *t*(621) = − 3.51, *p* < .001; intake of medication: *t*(621) = 1.76, *p* = .08; age: *r*(623) = − .01, *p* = .10; IQ score *r*(623) = − .10, *p* = .02) (see Table 1). Women (*M* = 10.30, *SD* = 5.89) scored higher than men (*M* = 8.63, *SD* = 6.04) as did participants with medication (*M* = 10.02, *SD* = 6.24) compared to those without (*M* = 9.15, *SD* = 5.85). Hence, all covariates were retained and entered into the first block of the HMLR models.

The combination of covariates and predictors specified in the first HMLR model presented in Table 3 significantly predicted the severity of insomnia symptoms, explaining 8% of the variance seen in ISI scores (*R*^2^ = .08, *F*(6, 504) = 7.49, *p* < .001). Squared partial correlation coefficients obtained for the first block including the covariates suggested the biggest contribution of sex (*β* = .16, *t*(506) = 3.49, *p* = .001, *sr*^2^ = .02) followed by IQ (*β* = − .09, *t*(506) = − 2.08, *p* = .04, *sr*^2^ = .01), whereas the other covariates did not reach significance. Introducing SPQ total and AQ social skill scores into the model led to an 4% increase in the amount of variance explained, which was statistically significant (*R*^2^*∆* = .04, *F*(2, 504) = 12.18, *p* < .001). Both main scores uniquely explained 2% of the variance in ISI scores. Obtained beta weights indicated higher insomnia symptom severity in individuals with higher sensory reactivity (*β* = − .14, *t*(504) = − 3.18, *p* = .002, *sr*^2^ = .02) and lower social skills (*β* = .15, *t*(504) = 3.32, *p* = .001, *sr*^2^ = .02).

**Table 3:** Outcome of the first hierarchical multiple linear regression analysis predicting insomnia severity

Covariates/predictors	*β*	*t*	*p*	*sr* ^2^	*R*	*R* ^2^	*R* ^2^ *∆*
Block 1: covariates					.19	.04	.04
Medication intake	− .06	− 1.36	.17	.003			
Sex	.16	3.49	.00	.023			
Age	− .00	.06	.95	.000			
IQ	− .09	− 2.08	.04	.008			
Block 2: predictor					.29	.08	.04
SPQ total score	− .15	− 3.16	.00	.018			
AQ-28 social skills	.15	3.32	.00	.020			

*R*^2^ = .08, *F*(6, 504) = 7.49, *p* < .001; Beta’s are standardized beta’s for the full model, i.e. the value of the beta when all covariates and predictors were included. *R*^2^*∆* refers to the difference between the model including covariates only (block 1) and the model entailing the predictors (block 2) as well

*SPQ* Sensory Perception Quotient

Based on the finding of a significant effect of overall sensory reactivity on insomnia symptom severity, a second HMLR model aimed at disentangling the individual contribution of the five main sensory modalities. Replacing the SPQ total score by subscale values for smell, vision, hearing, touch, and taste (see Table 4 for a summary) led to an increase of 7% in the variance of insomnia severity scores explained compared to a model relying on the covariates only (*R*^2^*∆* = .07, *F*(6, 500) = 6.89, *p* < .001), resulting in to a total *R*^2^ of .11 (*F*(10, 500) = 6.24, *p* < .001). Note, that this is slightly higher than the value of the first HMLR model, which was only able to account for 8% of the variance in insomnia scores. In line with our hypotheses, visual reactivity significantly predicted insomnia severity with higher insomnia scores in individuals with higher visual reactivity (*β* = − 0.27, *t(*500*)* = − 4.34, *p* < .001, *sr*^2^ = .03). This factor could uniquely account for 3% of the variance observed. Further, the effect of social skills remained significant (*β* = .13, *t(*500*)* = 2.96, *p* = .003, *sr*^2^ = .02). None of the other sensory modalities reached significance.

**Table 4:** Outcome of the second hierarchical multiple linear regression analysis predicting insomnia severity

Covariates/predictors	*β*	*t*	*p*	*sr* ^2^	R	*R* ^2^	*R*^2^∆
Block 1: covariates					.19	.04	.04
Medication intake	− .06	− 1.36	.17	.003			
Sex	.16	3.49	.00	.023			
Age	− .00	.06	.95	.000			
IQ	− .09	− 2.08	.04	.008			
Block 2: predictors					.33	.11	.07
SPQ smell	− .02	− .31	.76	.000			
SPQ vision	− .27	− 4.34	.00	.033			
SPQ hearing	.06	.96	.34	.002			
SPQ touch	− .03	− .38	.71	.000			
SPQ taste	.06	.96	.34	.002			
AQ-28 social skills	.13	2.96	.00	.016			

*R*^2^ = .11; *F*(10, 500) = 6.24, *p* < .001; Beta’s are standardized beta’s for the full model, i.e. the value of the beta when all covariates and predictors were included. *R*^2^*∆* refers to the difference between the model including covariates only (block 1) and the two models entailing the predictors (block 2) as well

*SPQ* Sensory Perception Quotient

## Discussion

The present study aimed to advance the understanding of potential mechanisms underlying insomnia in adults with ASD by examining the relationship of insomnia severity with sensory hyper-reactivity and social skills. In line with the assumption that sleep problems in ASD are not limited to childhood but persist throughout development, the average ISI score within the present sample was indicative of moderate insomnia. The severity of these symptoms was predicted by the overall sensory responsivity as well as the social skills of participants. Specifically, higher sensory hyper-reactivity and lower social skills were associated with more severe insomnia, which is in close resemblance to findings in pediatric populations based on parent or caregiver reports (Liu et al. 2006; Mazurek and Petroski 2015; Reynolds et al. 2011; Tzischinsky et al. 2018) and supports the notion that faulty entrainment of the circadian system associated with reduced sensory and social input could contribute to poor sleep in adults with ASD (Baker and Richdale 2017; Bourgeron 2007; Patzold et al. 1998; Richdale and Schreck 2009). Interestingly, the effect of sensory reactivity seemed to be driven by the visual domain which was the only significant sensory predictor in a second analysis assessing the effect of the modalities smell, vision, hearing, touch, and taste. This restriction to the visual modality nicely corresponds with the importance of photoperiodic information for the entrainment of circadian rhythms and, thus, can be seen as further evidence for the circadian dysregulation hypothesis. The absence of effects in the remaining modalities on the other hand may imply that hyper-reactivity in non-visual modalities does not reduce the availability of photoperiodic information and social cues and hence does not contribute to sleep problems significantly.

Diverging from these conclusions, some authors have proposed a more direct link between overall sensory hyper-reactivity and insomnia in ASD arguing that sleep problems may result from strong physiological reactions in response to slight, seemingly benign changes in the sleeping environment, such as unfamiliar noises or temperature differences (e.g. Reynolds et al. 2011). Based on the results of our second HLMR this scenario seems unlikely. Significant effects were restricted to the visual domain, yet there is no reason to believe that disturbing stimulation during bedtime should be purely visual. Unfortunately, the possibility to compare these results remains limited, as, to the best of our knowledge, only two studies have investigated the unique contribution of different sensory modalities instead of relying on composite scores. Both Hollway et al. (2013) and Tzischinsky et al. (2018) examined the effect of sensory reactivity measured by means of the Short Sensory Profile (SSP; Dunn 1999) and an adapted Hebrew version (Neuman et al. 2004), respectively, on sleep problems in a pediatric sample. While sleep problems were predicted by reactivity in the auditory, oral and olfactory domain in the first study, touch and oral sensitivities were significant predictors in the second study, leaving room for speculation why an effect of the visual domain was found in the present study. From a methodological perspective, the reliance on parent reports may have diminished the accuracy of sensitivity estimates analyzed in both studies, as sensory perception is highly subjective and may not always results in observable behaviors. Moreover, age discrepancies between the samples examined may account for differences in results highlighting the need for lifespan studies within this field.

The rather small effect sizes reported in the present study suggest that other factors not assessed in this study contribute to the emergence and persistence of sleep problems in adults with ASD, although the values obtained seem to lie within the normal outcome range of correlational sleep research (e.g. see Hollway et al. 2013; Nadeau et al. 2015). First, people with ASD are more likely to have comorbid depression and anxiety, due to overlapping diagnostic criteria and high genetic correlations between these three types of symptoms, indicating a shared genetic vulnerability (Hammerschlag et al. 2017; Jansen et al. 2018). Although no significant overall genetic correlation was found between insomnia and ASD (Hammerschlag et al. 2017), insomnia risk variants were found in a specific gene previously implicated in ASD (Jansen et al. 2018). Second, other core features of the ASD symptomatology such as difficulties handling deviations from daily routines could contribute to observed sleep problems and thus warrant further investigation. Closely related, hyperarousal (Bonnet and Arand 2010) is a key feature of insomnia. Hyperarousal may be perpetuated by difficulties maintaining consolidated Rapid Eye Movement sleep (Wassing et al. 2016), which has also been reported in ASD (Buckley et al. 2010; Diomedi et al. 1999). Insufficient activation of the subcortical caudate nucleus of the brain is considered key to hyperarousal (Stoffers et al. 2014) and has been consistently reported in fMRI studies in ASD (Turner et al. 2006). A recent post-mortem study in ASD (Adorjan et al. 2017) suggested involvement of reduced caudate interneuron density in the excitation/inhibition disbalance—also implicated in insomnia (Colombo et al. 2016a, b; Van der Werf et al. 2010). Next to the involvement of subcortical structures, individual differences in brain connectivity have also been implicated in one’s typical quality of sleep. A lower white matter integrity in the inferior fronto-occipital fascicle is associated both with worse sleep quality in healthy adults (Piantoni et al. 2013) and with the deficits of sensory processing in ASD (Pryweller et al. 2014).

Thus, future studies using circadian rhythm assessment and fMRI are needed to more directly address whether insomnia symptoms in ASD involve the biological clock and rhythms in e.g. body temperature (Scheer et al. 2005) and activity levels (Van Someren et al. 1998) and/or other factors. In addition, scheduled social interaction and its effect on sleep could be assessed using the Social Rhythm Metric Diary (Monk et al. 1992). Bright light avoidance in everyday life could be monitored with miniature sensors (Itzhacki et al. 2018), a method that has been applied successfully in insomnia research (Te Lindert et al. 2018).

### Limitations

The conclusions that can be drawn based on the results reported are limited by the cross-sectional nature of the study as well as the absence of (an) appropriate control group(s) including typically developing individuals and/or other clinical populations. Thus, the question whether the patterns revealed are ASD specific or a common feature shared, for example, with other developmental disorders remains unexplored and an important area for future inquiries. With respect to the measures applied, the usage of online questionnaires may introduce a bias by impeding the participation of non-verbal and non-literate adults. Furthermore, the lack of objective sleep assessment instruments such as actigraphy and polysomnography may be criticized. To date, studies investigating the ASD sleep phenotype by means of these measures have, however, produced rather mixed results with respect to discrepancies from typically developing populations despite elevated subjective complaints in subjects with ASD (Tani et al. 2005). Thus, subjective reports remain a valuable source of information. Closely related, the present study did not include a measurement of melatonin concentrations. Thus, it did not directly assess circadian dysregulations. Likewise, our questionnaires did not specifically address the availability of sleep related interventions such as blue light therapy, melatonin administration or behavioral therapies. Thus, we cannot evaluate which effect presence or absence of these therapies has on the results observed.

## Conclusions and Clinical Implications

Overall, the present study showed that insomnia severity in ASD is associated with sensory reactivity, especially in the visual domain, and impaired social skills. The findings are indirectly compatible with the notion that limited entrainment of the circadian system caused by reduced sensory and social input could contribute to poor sleep in adults with ASD. Consequently, interventions targeting sensory reactivity, social skills and scheduled entrainment could attenuate the observed problems. Other options include behavioral interventions involving sleep timing as well as the administration of melatonin for which favorable effects have been demonstrated in children with ASD (Cortesi et al. 2010; Delemere and Dounavi 2018). More detailed studies are needed, however, to address whether a suboptimal functioning of the biological clock underlies these associations and to identify further factors which could contribute to observed sleep problems.
